# Recent Advances in Endoscopic Ultrasound for Gallbladder Disease Diagnosis

**DOI:** 10.3390/diagnostics14040374

**Published:** 2024-02-08

**Authors:** Kosuke Takahashi, Eisuke Ozawa, Akane Shimakura, Tomotaka Mori, Hisamitsu Miyaaki, Kazuhiko Nakao

**Affiliations:** Department of Gastroenterology and Hepatology, Graduate School of Biomedical Sciences, Nagasaki University, Nagasaki 852-8501, Japan; eisukeozawa@nifty.com (E.O.); tomotakamori1021@nagasaki-u.ac.jp (T.M.); hmiya0629@yahoo.co.jp (H.M.); kazuhiko@nagasaki-u.ac.jp (K.N.)

**Keywords:** gallbladder disease, lesions, endoscopic ultrasound, artificial intelligence

## Abstract

Gallbladder (GB) disease is classified into two broad categories: GB wall-thickening and protuberant lesions, which include various lesions, such as adenomyomatosis, cholecystitis, GB polyps, and GB carcinoma. This review summarizes recent advances in the differential diagnosis of GB lesions, focusing primarily on endoscopic ultrasound (EUS) and related technologies. Fundamental B-mode EUS and contrast-enhanced harmonic EUS (CH-EUS) have been reported to be useful for the diagnosis of GB diseases because they can evaluate the thickening of the GB wall and protuberant lesions in detail. We also outline the current status of EUS-guided fine-needle aspiration (EUS-FNA) for GB lesions, as there have been scattered reports on EUS-FNA in recent years. Furthermore, artificial intelligence (AI) technologies, ranging from machine learning to deep learning, have become popular in healthcare for disease diagnosis, drug discovery, drug development, and patient risk identification. In this review, we outline the current status of AI in the diagnosis of GB.

## 1. Introduction

Gallbladder (GB) diseases are relatively common and include various lesions, such as gallstones, cholesterol polyps, adenomyomatosis, and GB carcinoma. Transabdominal ultrasonography (TUS) is widely used as a screening test for GB lesions because it is less invasive and can be performed easily. Screening using TUS is also useful for early detection of GB carcinoma, with a detection rate of carcinoma in the general Japanese population of 0.011% [1]. Recent advances in TUS technology have enabled high-frequency transducers to provide resolutions greater than 8 cm, covering the entire GB.

Endoscopic ultrasonography (EUS) has high spatial resolution. It allows detailed examination of the GB, especially the cystic duct and neck of the GB, because it scans from within the digestive tract, allowing for observation with a higher resolution. This high spatial resolution makes it possible to diagnose lesions qualitatively. Recently, contrast-enhanced harmonic EUS (CH-EUS), which provides an evaluation of blood flow, has enabled a more accurate diagnosis of GB tumors than fundamental B-mode EUS [2,3]. Furthermore, EUS-guided fine-needle aspiration (EUS-FNA) is useful for obtaining tissue samples from GB tumors for pathological evaluation [4,5].

Deep learning (DL) is an artificial intelligence (AI) algorithm and an advanced machine learning method that uses neural networks [6]. Remarkable advances have recently been made in increasing the efficacy of AI and reducing the costs associated with the diagnosis of various lesions [7,8]. DL is an informative medical tomography method that can help in the early diagnosis of GB based on US images.

This review describes recent advances in the diagnosis of GB lesions, with a focus on EUS and related technologies.

## 2. Differential Diagnosis of GB Disease

In ultrasonography, the wall of the GB is visualized as two layers: a hypoechoic inner layer and a hyperechoic outer layer, which correspond to the mucosa through the shallow and deep subserosal layers, respectively [9]. Normally, the GB wall is at most 3 mm thick and has a smooth luminal surface.

GB lesions were classified as wall thickening or protuberant GB lesions [10,11]. We outline the differential diagnosis and EUS characteristics of these two types of lesions.

### 2.1. GB Wall-Thickening Lesions (Table 1)

GB wall-thickening includes GB disorders such as adenomyomatosis, chronic cholecystitis, hyperplasia associated with pancreaticobiliary malfunction, IgG4-related disease, and GB carcinoma [12,13,14,15]. Therefore, distinguishing early-stage carcinoma from benign wall thickening of the GB is important [16]. The contour of the lesion, wall thickness patterns, intramural cystic space, and GB wall enhancement patterns were used as differential points.

The main types of GB wall-thickening lesions are as follows:

(i) Adenomyomatosis

**Figure 1:**
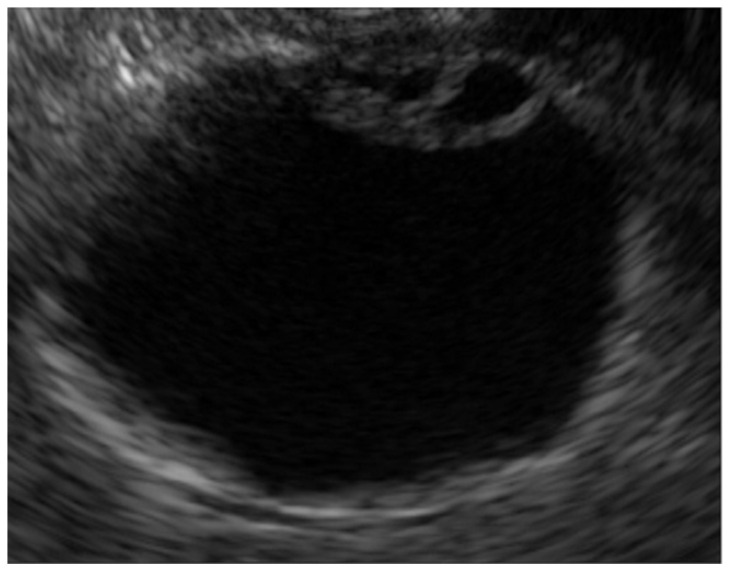
Adenomyomatosis. Rokitansky-Aschoff sinuses are visualized as small cystic spaces within the thickened GB wall.

A key point in its diagnosis is to confirm the presence of cystic anechoic spots that reflect the Rokitansky-Aschoff sinuses (RAS) within the thickened wall (Figure 1) [17]. Comet-tail artifacts are also occasionally observed due to multipath reflections from the RAS or intramural calculi. Microcysts and comet tail artifacts reflecting RAS are generally not observed in GB carcinoma [18]. However, we must carefully check for irregular unevenness on the surface of adenomyomatosis in the US due to the possible coexistence of cancer [19].

(ii) Chronic cholecystitis, Xanthogranulomatous cholecystitis

**Figure 2:**
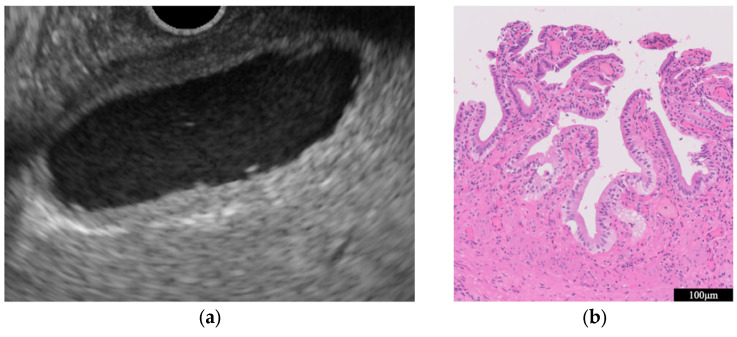
Chronic cholecystitis. The GB wall is depicted with uniform circumferential thickening while preserving the wall structure (**a**). Histopathologic findings included inflammatory cell infiltration of mainly lymphocytes and GB epithelium without atypical cells (**b**).

Chronic cholecystitis is characterized by chronic inflammation of the GB. EUS shows a uniform circumferential thickening of the GB wall with the preservation of its structure (Figure 2a) (Table 1) [20]. Histopathological characteristics include fibrosis, subserosal inflammatory cell infiltration, and muscularis propria thickening (Figure 2b).

**Figure 3:**
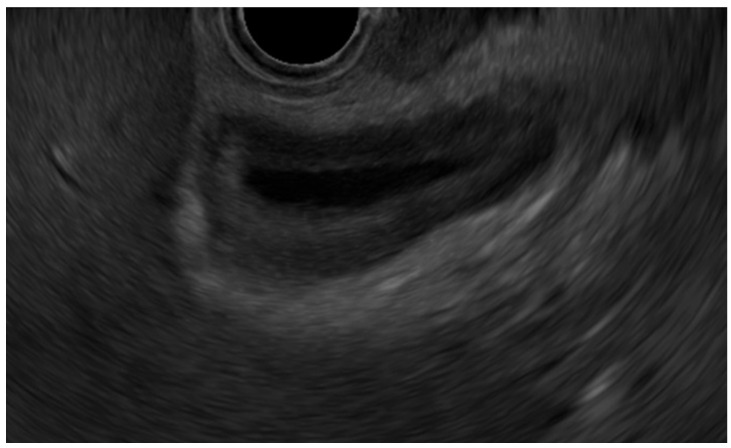
Immunoglobulin G4-related sclerosing cholecystitis. EUS shows GB wall thickening with smooth mucosal and homogenously thickened outer layer.

Xanthogranulomatous cholecystitis is a rare form of cholecystitis, and its differentiation from GB carcinomas is frequently problematic. The EUS findings of XGC showed that hypoechoic nodules were observed in 15–73% of patients, hypoechoic bands in 19%, and intermediate or varied hypoechoic shapes in 35% [21].

(iii) Immunoglobulin G4-related sclerosing cholecystitis (IgG4-CC) (Figure 3)

**Figure 4:**
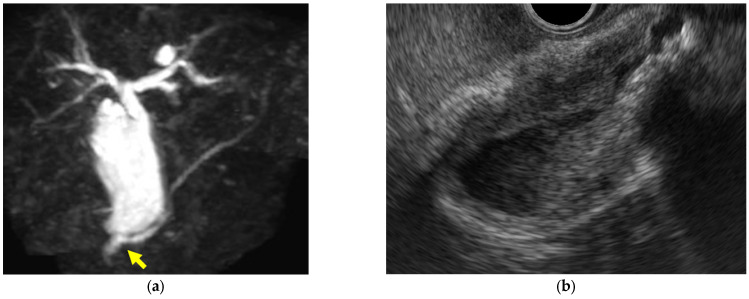
Mucosal hyperplasia associated with pancreaticobiliary maljunction. (**a**) MRCP shows the abnormal junction of the pancreatic duct and common bile duct (arrow). (**b**) EUS shows that the hypoechoic layer within the wall is markedly thickened.

IgG4-related cholecystitis, a manifestation of IgG4-related disease in GB, remains a clinical problem, with focal wall thickening that mimics GB carcinoma in EUS [14,22,23,24]. The presence of wall thickening with an intact or smooth mucosal layer, followed by a homogeneously thickened outer layer, is helpful in distinguishing IgG4-CC from GB carcinoma [25].

(iv) GB mucosal hyperplasia associated with pancreaticobiliary maljunction (PBM) (Figure 4)

PBM is a congenital malformation in which the pancreas and bile ducts are anatomically joined outside the duodenal wall, and the mechanism of mucosal transformation of the GB from hyperplasia to cancer has been postulated. Hyperplasia of the GB mucous membrane is observed in 38% to 63% of patients with PBM, with an even higher rate of 90% to 100%, particularly in those without bile duct dilatation [26].

(v) - GB carcinoma

The morphology of GB carcinomas varies from elevated to wall-thickening. In the wall-thickening type, differentiation from adenomyomatosis and chronic cholecystitis is problematic. However, in GB carcinoma, the mucous membrane is irregular or papillated, the thickened areas do not have a uniform thickness, and the layered structure is not well-defined. The characteristic findings of malignant GB disease in EUS include wall thickening (>10 mm), hypoechoic internal echogenicity, internal echo patterns, and disrupted wall layering [27]. Kim et al. reported that GB wall thickening of >10 mm and hypoechoic internal echogenicity were independent predictive factors for neoplastic GB wall thickening [28]. However, the differentiation of malignant lesions from benign thickening of the GB wall remains challenging.

### 2.2. Protuberant Lesions (PLs) (Table 2)

PLs are focal elevations or protrusions that can be distinguished from the surrounding mucosa [29,30]. PLs are divided into two types (i.e., nonneoplastic and neoplastic) and should be differentiated from other benign lesions, such as cholesterol and hyperplastic polyps [30]. The differential diagnosis of neoplastic and nonneoplastic lesions is based on size, number, morphology, surface contour, internal echotexture, and internal structure. Classifying them into pedunculated or sessile (broad based) types is important. Most pedunculated lesions are benign, and cholesterol polyps are the most common.

Several studies have evaluated the use of EUS for the differential diagnosis of PLs [17,31]. Cho et al. focused on relatively hypoechoic areas in the polyp cores and reported that such hypoechoic cores in EUS are a strong predictive factor for neoplastic polyps [32]. The overall accuracy of EUS in differentiating neoplastic lesions from nonneoplastic lesions was 86.5% to 97% [33,34].

The main types of PLs are as follows:

(i) Nonneoplastic lesions (GB polyps)

**Figure 5:**
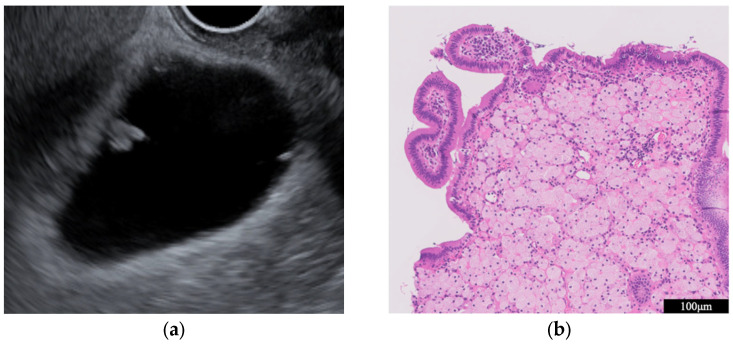
Cholesterol polyp. (**a**) EUS shows a pedunculated lesion with a granular surface. (**b**) A microscopic image (HE stain, intermediate power) shows that foam cells proliferate and form sheet-like aggregates within the subepithelial stroma.

The characteristic findings of cholesterol polyps in EUS are a deeply notched granular surface and morphology (Figure 5) [35]. The internal echo was rough or granular, and highly echogenic punctiform foci reflecting cholesterol were visible.

The findings of endoscopic ultrasound (EUS) of hyperplastic polyps show that they are papillary to lobulated and internally homogeneous.

(ii) Neoplastic lesions

**Figure 6:**
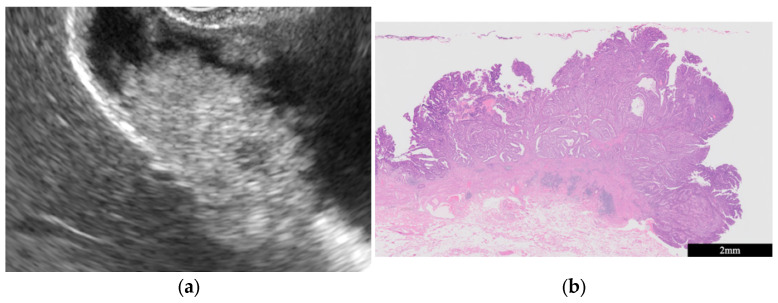
GB carcinoma. (**a**) EUS showing a 30 mm irregular sessile lesion at the fundus of the GB. (**b**) Pathological diagnosis was a moderately differentiated adenocarcinoma with invasion into the muscularis propria.

EUS shows an adenoma as a homogeneously isoechoic pedunculated or subpedunculated mass with a nodular or relatively smooth surface and an adenocarcinoma (pedunculated type) as a heterogeneously echogenic pedunculated mass with a nodular or smooth surface (Figure 6) [35]. Because the layered structure can be examined in detail using EUS, sessile lesions with a deep hypoechoic area or thinning of the hyperechoic outer layer can be diagnosed as GB carcinomas with a subserosal depth of invasion. However, in cases where the hyperechoic outer layer is retained, the depth of invasion may extend to the mucous membrane, muscularis, or shallow subserosal layer, depending on the case, and differentiation is difficult even using EUS.

## 3. Comparison of EUS and Other Modalities in the Detection of GB Lesions

In GB screening, the management of artifacts such as noise, reverberation, side lobes, and beam width is important. Noise and reverberation can cause coarse-grained echoes to appear, making it difficult to observe fundus lesions, which can lead to oversight. To manage these conditions, reducing gain settings and using high-resolution US (HRUS) may be helpful. HRUS is a technique that uses both low- and high-frequency transducers during evaluation. Compared to conventional US, HRUS can precisely delineate the layer structure of the GB wall. Several studies have reported little difference between HRUS and EUS’s ability to diagnose GB lesions (Table 3). Lee et al. [36] reported that the sensitivity, specificity, PPV, and NPV for the differential diagnosis of GPLs were 80%, 80%, 86%, and 73%, respectively, using HRUS, and 73%, 85%, 88%, and 69% using EUS. Jang et al. [37] found that HRUS and EUS sensitivity and specificity for the diagnosis of malignant neoplasms were 89.6%, 86.9%, and 86.9% and 86.2%, respectively.

However, the degree of frequency of the transducer for US scanning depends on the distance between the target organ and the transducer. On TUS, the fundus of the GB is often close to the abdominal wall, facilitating the use of high-frequency transducers, while the neck is far from the abdominal wall, requiring low-frequency transducers. This positioning causes side lobe artifacts, which can easily lead to overdiagnosis. In contrast, the EUS transducer is closer to the neck of the GB and cystic duct than the TUS transducer because it scans from within the digestive tract, allowing observation with a higher resolution. In contrast, the position of the GB fundus varies greatly between individuals, and caution is necessary, as failure to determine the overall structure of the GB before performing the examination may lead to oversight of the lesion and inability to obtain accurate observations.

CT is a useful adjunct to ultrasound in difficult or complicated forms of cholecystitis (emphysematous, gangrenous, or chronic cholecystitis). It is also useful for the preoperative evaluation of gallbladder cancer (anatomical extension to blood vessels, lymph nodes, liver parenchyma, and distant metastases). The sensitivity of CT for evaluating the gallbladder wall is excellent, but is less so for the assessment of the gallbladder contents. Furthermore, CT is suboptimal for spatial resolution and hence limited in its ability to provide a differential diagnosis of gallbladder lesions. Also, radiolucent stones and radiation exposure are clear limitations. EUS has been considered to be superior to CT in terms of GB imaging because it demonstrates the layered structures of GB and provides high-resolution images [34]. On the other hand, EUS has some limitations such as sedation, excessive manpower, and considerable time.

As mentioned above, EUS has several advantages over other imaging modalities in the diagnosis of GB lesions. On the other hand, EUS is estimated to require a long learning curve, including the acquisition of both technical and cognitive skills, which may limit its use in some countries and centers. Therefore, one of the future challenges is to lead with those countries and centers regarding the implementation of EUS.

## 4. EUS Scope

There are two types of EUS scope: radial scan (RS) and curved linear array (CL). These devices provide different images and are used in various visualization methods. Kaneko et al. [38] conducted a prospective comparative study of the different imaging capabilities of these two devices in the pancreaticobiliary region. CL-EUS was inferior to RS-EUS in terms of longitudinal GB visualization. However, there was no significant difference in the detection of GB lesions between the two groups. Confirmation using different modalities, such as CT, is important prior to EUS to minimize oversight during testing. As the structure and position of the GB vary from patient to patient, the curvature of the GB and the positions of the lesions should be verified in advance to avoid oversight during EUS.

Tips for GB examination for each type of EUS are shown below.

(i) RS-EUS

**Figure 7:**
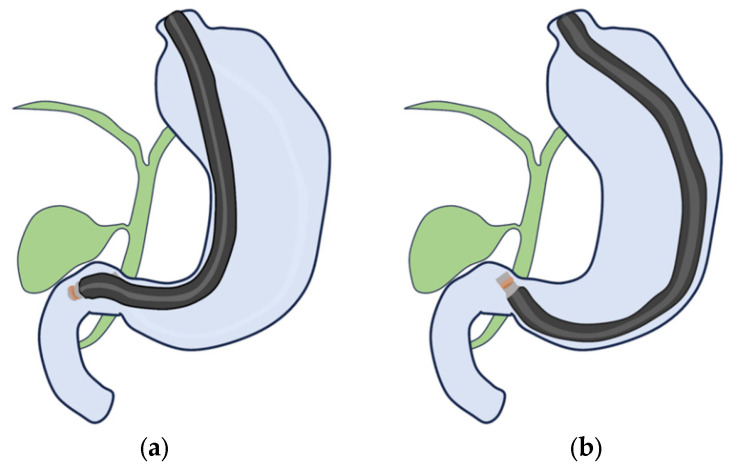
Radial scan type. (**a**) Short-scope position. (**b**) Long-scope position.

The GB is observed through the duodenum. First, the scope is advanced into the descending duodenum and extended to a short scope position (Figure 7a). The balloon is slightly inflated and withdrawn in counterclockwise rotation while checking the bile duct and then from the cystic duct to the neck of the GB. If the entire GB cannot be observed in the short-scope position, the tip of the scope is pressed against the superior duodenal curvature in the duodenal bulb to achieve the long-scope position (Figure 7b). The scope is advanced in the descending duodenal direction using a clockwise rotation. After the cystic duct is identified, the GB is observed sequentially from the neck to its base. The direction of the GB in the long-scope position is opposite to the short-scope position.

(ii) CL-EUS

**Figure 8:**
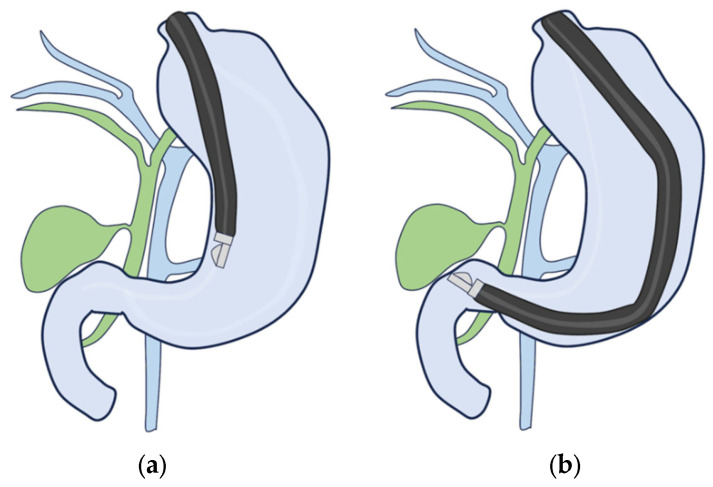
Curved linear array. (**a**) Transgastric scanning. (**b**) Duodenal bulb scanning.

The bile ducts are first identified using the portal vein as a landmark to observe the GB using transgastric scanning (Figure 8a). After the cystic duct is detected in the bile duct, the neck of the GB is identified by rotating the scope. However, it is not always easy to observe the entire GB from within the stomach.

The portal vein is identified by scanning the duodenal bulb (Figure 8b), and the bile and cystic ducts are detected by tilting the scope downward while pulling it counterclockwise. Rotating the scope while tracing the cystic ducts allows for visualization of the neck of the GB to the fundus.

Because the direction of rotation differs between patients, it is important to visualize the ducts sequentially from the cystic duct to the neck of the GB and to observe the GB fully, including its fundus.

## 5. Contrast Harmonic Imaging (CH-Imaging)

(i) CH-TUS

The evolution of diagnostic ultrasound (US) equipment has been remarkable. B-mode tomography was developed in the early 1980s, followed by the evaluation of blood flow in lesions using color and power Doppler methods in the late 1980s. Subsequently, CH-TUS using first-generation contrast agents (Levovist (Schering AG, Berlin, Germany)) was introduced in the late 1990s [39,40]. However, CH-TUS with first-generation contrast agents requires imaging via microbubble collapse using strong sound pressure. However, second-generation contrast agents can only be evaluated at low sound pressures. CH-TUS with second-generation contrast agents makes it easier to detect the target because the microbubbles are not destroyed by continuous observation using low sound pressure [41,42,43]. Previous studies have examined the utility of Sonazoid-based CH-TUS in the characterization of the liver [44,45] and GB lesions [9,46,47,48,49,50,51,52,53]. Some studies have shown that CH-TUS can overcome the limitations associated with conventional TUS and increase the diagnostic accuracy of GB diseases (the accuracy of CH-TUS and conventional TUS was 95.2% and 88.6%, respectively) [47,52]. Conventional TUS combined with CE-TUS can significantly improve the diagnostic accuracy of GB diseases and provide complementary diagnostic information for CT and MRI [54,55].

(ii) CH-EUS (Table 4)

As mentioned above, while the fundus of the GB is close to the abdominal wall and can be evaluated using high-resolution TUS, it is often difficult to observe lesions in the cystic duct and neck of the GB that are far from the abdominal wall. Thus, CH-EUS is a useful approach that addresses the limitations of CH-TUS. The usefulness of CH-EUS in the diagnosis of pancreatic [56,57,58,59,60,61], gastrointestinal [62,63], and GB lesions [9,46,47,48,49,50,51,52,53] has been reported.

**Table 4:** Studies within the last 10 years on the diagnostic performance of CH-EUS for GB lesions.

Author	Year	Patient	Study Design	Contrast Agent	Sensitivity	Specificity
Park, C.H et al. [64]	2013	20	retrospective	SonoVue	0.75	0.67
Choi et al. [65]	2013	90	retrospective	SonoVue	0.94	0.93
Imazu et al. [66]	2014	36	retrospective	Sonazoid	0.90	0.98
Sugimoto et al. [67]	2016	24	retrospective	Sonazoid	1.00	0.94
Kamata et al. [3]	2017	125	retrospective	Sonazoid	0.90	0.98
Leem et al. [68]	2018	145	retrospective	SonoVue	0.97	0.55

CH-EUS is useful for diagnosing GB wall thickening. Imazu et al. reported that CH-EUS showed significantly superior specificity and precision compared to conventional EUS in the diagnosis of malignant wall thickening of the GB [66]. They reported that the general sensitivity, specificity, and precision of EUS and CH-EUS for diagnosing malignant thickening of the GB wall were 83.3% vs. 89.6%, 65% vs. 98% (*p* < 0.001), and 73.1% vs. 94.4% (*p* < 0.001), respectively. Kamata et al. [3] reported that irregular vessels characterize GB carcinoma in vascular images and heterogeneous enhancement in perfusion images. The sensitivity, specificity, and precision of contrast-enhanced harmonic CH-EUS for the diagnosis of carcinoma were 90%, 98%, and 96%, respectively.

Various studies on protuberant lesions have also been reported [3,64,65,66,67,68]. In GB polyps with a maximum diameter of at least 10 mm, irregular intratumoral vessels, and perfusion defects are reportedly characteristic findings of GB cancer on CH-EUS imaging. Perfusion patterns were classified as diffuse enhancement, perfusion defect, or no enhancement in a contrast-enhanced harmonic CH-EUS study by Choi et al. [65]. The vessels were classified as regular, irregular, or non-regular. The sensitivity and specificity for the diagnosis of malignant polyps with irregular intratumoral vessel patterns in CH-EUS were 90.3 and 96.6%, respectively, while those showing perfusion defects were 90.3 and 94.9%. For the diagnosis of malignant polyps, the sensitivity and specificity of CH-EUS were superior to conventional EUS (CH-EUS vs. conventional EUS: 93.5 and 93.2%, respectively, vs. 90.0 and 91.1%, respectively).

(iii) CH-imaging phase

For the examination of liver disease, the CH-US phases are commonly divided into two vascular phases, the arterial, and the portal phases, with the contrast effect on the liver parenchyma progressively being enhanced in the portal phase rather than in the arterial phase [69]. Portal venous flow was not observed in patients with GB disease. However, some reports have identified two vascular phases: arterial and venous [47,70]. In the arterial phase, the peak intensity is assessed 10–30 s after contrast administration, whereas the venous phase shows continuous or decreased enhancement 60–180 s after injection.

## 6. Cytology and Biopsy for GB Lesion

(i) Endoscopic Transpupillary Approach

**Figure 9:**
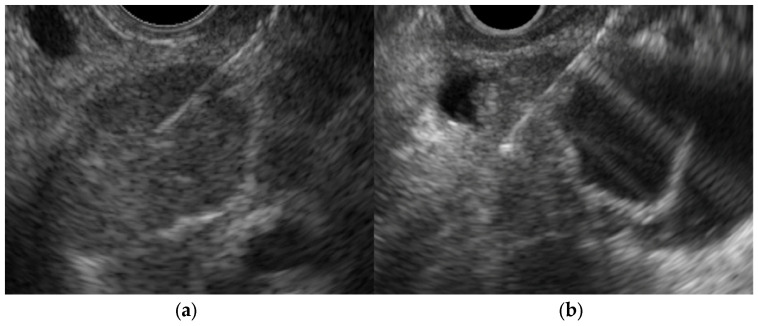
Endoscopic ultrasound-guided fine-needle aspiration/biopsy for malignant GB lesion. (**a**) Puncture of the enlarged lymph node. (**b**) Puncture of the GB wall-thickening lesions.

Pathological evaluation of GB disease is ideal for treatment decision making. However, it is difficult for anatomical reasons. The usefulness of cytological examination with an endoscopic transpapillary GB drainage tube has been reported [71]. Additionally, transpapillary GB biopsy has recently been reported using various new devices, such as biopsy forceps, large-diameter pusher catheters, cholangioscopes, and delivery systems [72,73,74,75,76]. Yane et al. [77] evaluated transpapillary GB biopsies using a newly designed device delivery system (Endosheather; Piolax Medical Device, Kanagawa, Japan). The success rate of selective insertion of the GB cannulation and delivery system was 90.9% (10/11). The success rate of the target lesion biopsy was 63.6% (7/11). However, transpapillary GB biopsy sometimes makes it difficult to obtain samples of lesions in the neck or body of the GB or elevated lesions of the GB for anatomical reasons. Furthermore, these methods are impossible in cases with a tortuous, thin cystic duct or failed GB cannulation. Therefore, cases are sometimes encountered in which a definitive diagnosis cannot be made pathologically, even with this method. In addition, these methods require highly skilled practitioners and present problems, such as perforation of the cystic duct when a guidewire is used.

(ii) EUS approach (Figure 9) (Table 5)

In recent years, EUS-FNA has been reported to be useful in obtaining sufficient tissue samples from various organs [57,78,79,80,81], such as GB mass lesions [82,83,84,85,86]. Hijioka et al. [4] reported that FNA can be performed on GB lesions without compromising diagnostic performance or safety. Furthermore, the diagnostic performance of EUS-FNA in GB lesions is high, with a sensitivity, specificity, and diagnostic precision of 80–100%, 100%, and 83–100%, respectively [82,83,87,88,89]. Furthermore, the staging of GB carcinoma is important for determining treatment. In particular, the presence of distant metastasis and para-aortic lymph node (PALN) metastasis are important factors in determining whether surgery is possible. PALN metastasis is classified as distant metastasis and is considered an unresectable factor by the Union for International Carcinoma Control (UICC) [90]. In some studies, EUS-FNA has been reported to be useful for the diagnosis of lymph nodes and PALN metastases [91]. Kurita et al. [92] reported that EUS-FNA had a higher sensitivity and specificity (96.7 and 100%, respectively) than positron emission tomography-CT (PET-CT) in the diagnosis of PALN metastasis. EUS-FNA is superior to PET/CT for the preoperative staging of PALN in patients with GB [91]. Although EUS-FNA has been considered to be a safe diagnostic modality with <1% of morbidity and mortality rates, it is not free from complications. The most common complications of EUS-FNA include infections, bleeding, and pancreatitis. Moreover, EUS-FNA are not recommended for resectable GB carcinoma, because this procedure may induce biliary peritonitis and would also cause peritoneal dissemination [93].

**Table 5:** Studies within the last 10 years on the diagnostic performance of EUS-FNA for GB lesions.

Author	Year	Patient	Study Design	Sensitivity	Specificity	Accuracy
Ogura et al. [89]	2014	16	retrospective	1.00	1.00	1.00
Singla et al. [86]	2018	101	retrospective	0.91	1.00	0.91
Goyal, S et al. [84]	2023	489	retrospective	-	-	0.95
Tong, T et al. [85]	2023	27	retrospective	0.95	1.00	0.96
Kuraishi et al. [94]	2023	187	retrospective	0.97	1.00	0.97

The basic approach involves puncturing the gallbladder lesion through the liver parenchyma to reduce the risk of bile leakage. Unlike TUS, EUS-FNA, which punctures the opposite side of the liver, may directly puncture the GB wall. In this case, the sample could be obtained relatively safely without penetrating the tumor through tangentially puncturing the gallbladder wall. Furthermore, the lesion in the gallbladder neck was closer to the EUS transducer than the TUS transducer, allowing for a more accurate puncture with EUS-FNA. Furthermore, the puncture of metastatic lymph nodes using TUS is generally difficult, but EUS-FNA allows for easy sample collection. Kuraishi et al. reported that a puncture targeting the liver invasion site significantly increased the diagnostic accuracy when sampling the primary mass in cases of malignancy. The GB is a mobile structure that poses difficulties for EUS-FNA, while the site of liver infiltration is rigidly fixed by the surrounding structures, making the puncture of this target easier and avoiding needle passage through the lumen of the GB. Furthermore, aggressive malignant cells that are more diagnostically relevant are more likely to be detected in the infiltrated portion. Therefore, they concluded that an approach targeting the invaded liver site could increase diagnostic yield and prevent complications caused by bile leakage [94].

Indications and strategies for the EUS-FNA of GB mass lesions should include the following:

1. When it is difficult to categorize a lesion as benign or malignant or when surgery is extremely invasive, EUS-FNA should be considered.
2. In patients with GB tumors accompanied by either liver invasion or metastasis or both, including lymph nodes, they should be first punctured.
3. For GB masses or wall thickening, the lesion should be punctured without the puncture needle passing through the lumen of the GB [2].

In EUS-FNA for pancreatic carcinoma, CH-EUS guidance is reported to be useful for obtaining pancreatic tissue [95]. For GB lesions, CH-EUS allows clear discrimination of the sludge of the GB tumors and the walls of the GB, allowing the GB tumors to be punctured while avoiding puncturing fluid spaces [96,97]. Therefore, the CH-EUS guide allows for appropriate positioning of the needle within the GB tumor through avoiding fluid space, which can lead to higher tissue volume [97]. Furthermore, the CH-EUS guidance for FNA has been reported to be useful in avoiding complications of GB, such as bile peritonitis and needle track seeding.

With the recent development of next-generation sequencing, the number of patients receiving individualized medicine based on genomic biomarkers has increased. GB and biliary tract carcinomas contain driver genes such as ERBB2, PIK3CA, IDH1/2, BRCA1/2, and FGFR2 fusion genes [98,99]. Next-generation sequencing is possible for tissues of GB carcinoma obtained using EUS-FNA. Therefore, in the future, EUS-FNA may become an essential examination when deciding on chemotherapy for GB carcinomas [100].

## 7. AI Approach to the Diagnosis of GB Lesions

**Figure 10:**
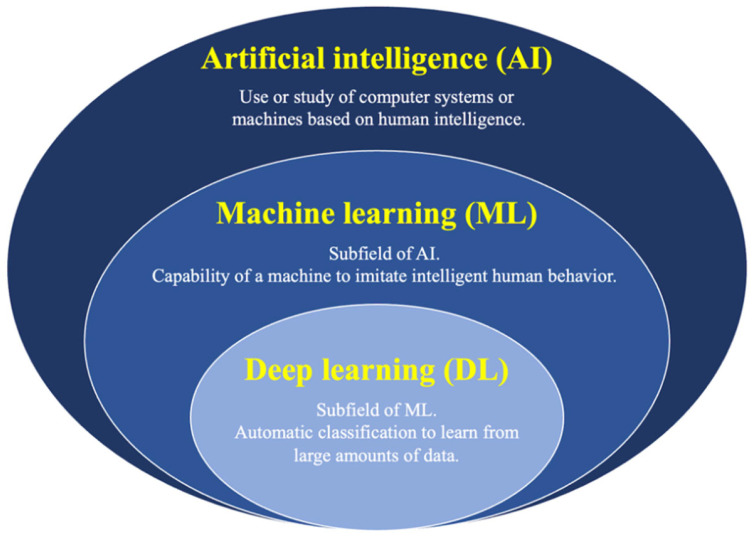
Primary concepts of artificial intelligence.

AI is a mathematical prediction technique that involves automated learning and recognition of data patterns. DL is an AI algorithm and an advanced machine-learning method that uses neural networks (Figure 10) [6]. Currently, AI techniques, ranging from machine learning (ML) to DL, are prevalent in healthcare for disease diagnosis, drug discovery, drug development, and patient risk identification [101,102,103,104,105,106,107,108,109,110]. Advances in DL have led to significant progress in the field of medical image analysis and understanding [111]. Moreover, with progress at the algorithmic level, the availability of high-performance computing machines, and large amounts of data, DL-based methods have become increasingly popular. DL models that use image input have generally been developed to classify lesions or no lesions, group lesion types, and detect lesions or segment lesions [112]. DL algorithms can assist analysts in the early identification, treatment, and recognition of diseases and subsequently provide efficient methods for medical diagnostics.

Regarding the diagnosis of GB lesions, a well-developed AI approach based on US images can increase the accuracy of the diagnosis of the disease [113]. Jang et al. [114] evaluated the diagnostic performance of an EUS-AI system with the ResNet50 architecture using an AI development cohort of 1039 EUS images (836 GB of polyps and 203 gallstones). Diagnostic performance was verified using an external validation cohort of 83 patients and was compared with the performance of EUS endoscopists. For the differential diagnosis of neoplastic and nonneoplastic GB polyps, sensitivity and specificity were 33.3% and 96.1% for EUS-AI and 74.2% and 44.9%, respectively, for endoscopists. They concluded that this newly developed AI system showed favorable performance in the diagnosis of neoplastic GB polyps, with a performance comparable to EUS endoscopists. Jeong et al. [115] evaluated the diagnostic performance of an AI system with Inception v3 architecture using a test dataset (*n* = 98). Using the test dataset, three radiologists with different levels of experience retrospectively graded the possibility of neoplastic polyps using a 5-point confidence scale. For the differential diagnosis of GB polyps using AI alone, the diagnostic performance was of 74.3% sensitivity, 92.1% specificity, and 85.7% accuracy. With AI assistance, the diagnostic specificity of the reviewers improved (65.1–85.7 to 71.4–93.7). They concluded that an AI-based decision-support system could be used as an assistant tool for reviewers.

Currently, there are some reports with strong evidence for the usefulness of AI for the diagnosis of GB disease. However, AI has the potential to be a breakthrough in the diagnosis of GB diseases, while other modalities have limited diagnostic capabilities.

## 8. Conclusions

Conventional and CH-EUS play several important roles, such as the detection of GB lesions and the differentiation between benign and malignant types. EUS-FNA is not only a useful examination for the diagnosis and staging of GB carcinoma but is also becoming an essential examination for determining the choice of chemotherapy. Additionally, remarkable advances in AI are expected to contribute to a more accurate diagnosis of GB lesions.

## Figures and Tables

**Table 1:** EUS features of GB wall-thickening lesions.

	Surface	Internal Echo	Layer Structure
Adenomyomatosis	Smooth	Multiple anechoic areas comet tail artifact	Preserved
Acute cholecystitis	Smooth	No distinctive findings	Preserved
Chronic cholecystitis	Smooth	No distinctive findings	Preserved
Xanthogranulomatous cholecystitis	Smooth	Mixed hyperechoic and hypoechoic echotexture	Irregular or disrupted
Immunoglobulin G4-related sclerosing cholecystitis	Smooth	No distinctive findings	Preserved
GB mucosal hyperplasia associated with pancreaticobiliary maljunction	Smooth	Uniform hypoechogenicity	Preserved
GB carcinoma	Irregular or papillated	Uneven hypoechogenicity	Irregular or disrupted

**Table 2:** EUS features of GB protuberant lesions.

	Form	Pedunculation	Surface	Internal Echo
Cholesterol polyp	Morular or oval	Pedunculated	Granular	Rough or granular Hyperechoic spots
Hyperplastic polyp	Papillated or lobulated	Pedunculated	Smooth	Uniform low echogenicity
Adenoma	Oval	Pedunculated or subpedunculated	Smooth or nodular	Homogenous isoechogenicity Multiple microcystic spaces
Carcinoma	Oval or irregular	Sessile > Pedunculated	Smooth or nodular	Heterogenous dense echogenicity Hypoechoic areas at the core

**Table 3:** Studies comparing HRUS and EUS in the detection of GB lesions.

Author	Year	Patient	Study Design	Diagnostic Modality	Sensitivity	Specificity	Accuracy
Lee et al. [36]	2009	144	retrospective	HRUS	0.80	0.80	0.80
				EUS	0.73	0.85	0.78
Jang et al. [37]	2017	125	retrospective	HRUS	0.90	0.87	0.88
				EUS	0.87	0.86	0.87

## Data Availability

Data supporting reported results can be found in the references at the end of this article.

## References

[1] Mihara S., Yoshioka R., Tamada M. (2005). Update of ultrasonographic screening for gallbladder cancer in mass survey (in Japanese). Tanto Sui.

[2] Hijioka S., Nagashio Y., Ohba A., Maruki Y., Okusaka T. (2021). The Role of EUS and EUS-FNA in Differentiating Benign and Malignant Gallbladder Lesions. Diagnostics.

[3] Kamata K., Takenaka M., Kitano M., Omoto S., Miyata T., Minaga K., Yamao K., Imai H., Sakurai T., Nishida N. (2018). Contrast-enhanced harmonic endoscopic ultrasonography for differential diagnosis of localized gallbladder lesions. Dig. Endosc..

[4] Hijioka S., Hara K., Mizuno N., Imaoka H., Ogura T., Haba S., Mekky M.A., Bhatia V., Hosoda W., Yatabe Y. (2012). Diagnostic yield of endoscopic retrograde cholangiography and of EUS-guided fine needle aspiration sampling in gallbladder carcinomas. J. Hepatobiliary Pancreat. Sci..

[5] Hijioka S., Mekky M.A., Bhatia V., Sawaki A., Mizuno N., Hara K., Hosoda W., Shimizu Y., Tamada K., Niwa Y. (2010). Can EUS-guided FNA distinguish between gallbladder cancer and xanthogranulomatous cholecystitis?. Gastrointest. Endosc..

[6] LeCun Y., Bengio Y., Hinton G. (2015). Deep learning. Nature.

[7] Mori Y., Kudo S.E., East J.E., Rastogi A., Bretthauer M., Misawa M., Sekiguchi M., Matsuda T., Saito Y., Ikematsu H. (2020). Cost savings in colonoscopy with artificial intelligence-aided polyp diagnosis: An add-on analysis of a clinical trial (with video). Gastrointest. Endosc..

[8] Wu L., Zhou W., Wan X., Zhang J., Shen L., Hu S., Ding Q., Mu G., Yin A., Huang X. (2019). A deep neural network improves endoscopic detection of early gastric cancer without blind spots. Endoscopy.

[9] Hashimoto S., Nakaoka K., Kawabe N., Kuzuya T., Funasaka K., Nagasaka M., Nakagawa Y., Miyahara R., Shibata T., Hirooka Y. (2021). The Role of Endoscopic Ultrasound in the Diagnosis of Gallbladder Lesions. Diagnostics.

[10] Okaniwa S. (2021). Everything you need to know about ultrasound for diagnosis of gallbladder diseases. J. Med. Ultrason 2001.

[11] Okaniwa S. (2021). How Can We Manage Gallbladder Lesions by Transabdominal Ultrasound?. Diagnostics.

[12] Yu M.H., Kim Y.J., Park H.S., Jung S.I. (2020). Benign gallbladder diseases: Imaging techniques and tips for differentiating with malignant gallbladder diseases. World J. Gastroenterol..

[13] Watanabe K., Kamisawa T., Chiba K., Kikuyama M., Nakahodo J., Igarashi Y. (2021). Gallbladder wall thickening in patients with IgG4-related diseases, with special emphasis on IgG4-related cholecystitis. Scand. J. Gastroenterol..

[14] Harada Y., Mihara K., Amemiya R., Nakagawa M., Hanada R., Inoue K., Shito M., Orikasa H., Aiura K. (2022). Isolated IgG4-related cholecystitis with localized gallbladder wall thickening mimicking gallbladder cancer: A case report and literature review. BMC Gastroenterol..

[15] Ganeshan D., Kambadakone A., Nikolaidis P., Subbiah V., Subbiah I.M., Devine C. (2021). Current update on gallbladder carcinoma. Abdom. Radiol..

[16] Miyoshi H., Inui K., Katano Y., Tachi Y., Yamamoto S. (2021). B-mode ultrasonographic diagnosis in gallbladder wall thickening. J. Med. Ultrason. 2001.

[17] Tanaka K., Katanuma A., Hayashi T., Kin T., Takahashi K. (2021). Role of endoscopic ultrasound for gallbladder disease. J. Med. Ultrason. 2001.

[18] Shapiro R.S., Winsberg F. (1990). Comet-tail artifact from cholesterol crystals: Observations in the postlithotripsy gallbladder and an in vitro model. Radiology.

[19] Nabatame N., Shirai Y., Nishimura A., Yokoyama N., Wakai T., Hatakeyama K. (2004). High risk of gallbladder carcinoma in elderly patients with segmental adenomyomatosis of the gallbladder. J. Exp. Clin. Cancer Res..

[20] Päivänsalo M., Siniluoto T., Myllylä V., Kairaluoma M.I., Kallioinen M. (1987). Ultrasound in acute and chronic cholecystitis. RoFo.

[21] Kim P.N., Ha H.K., Kim Y.H., Lee M.G., Kim M.H., Auh Y.H. (1998). US findings of xanthogranulomatous cholecystitis. Clin. Radiol..

[22] Lee Y.C., Chon H.K., Choi K.H. (2022). IgG4-related sclerosing cholangitis involving the gallbladder mimicking a hilar cholangiocarcinoma. Endoscopy.

[23] Ichinokawa M., Matsumoto J., Kuraya T., Kuwabara S., Wada H., Kato K., Ikeda A., Murakawa K., Ono K. (2019). A rare case of localized IgG4-related sclerosing cholecystitis mimicking gallbladder cancer. J. Rural Med..

[24] Zhang R., Lin H.M., Cai Z.X., Du S.J., Zeng H., Xu L.B., Wang J., Liu C. (2020). Clinical strategies for differentiating IgG4-related cholecystitis from gallbladder carcinoma to avoid unnecessary surgical resection. Sci. China Life Sci..

[25] Kuwatani M., Sakamoto N. (2021). Clinical and Image Characteristics of IgG4-Related Sclerosing Cholecystitis. Diagnostics.

[26] Tsuchida A., Itoi T., Endo M., Kitamura K., Mukaide M., Itokawa F., Ozawa T., Aoki T. (2004). Pathological features and surgical outcome of pancreaticobiliary maljunction without dilatation of the extrahepatic bile duct. Oncol. Rep..

[27] Mizuguchi M., Kudo S., Fukahori T., Matsuo Y., Miyazaki K., Tokunaga O., Koyama T., Fujimoto K. (1997). Endoscopic ultrasonography for demonstrating loss of multiple-layer pattern of the thickened gallbladder wall in the preoperative diagnosis of gallbladder cancer. Eur. Radiol..

[28] Kim H.J., Park J.H., Park D.I., Cho Y.K., Sohn C.I., Jeon W.K., Kim B.I., Choi S.H. (2012). Clinical usefulness of endoscopic ultrasonography in the differential diagnosis of gallbladder wall thickening. Dig. Dis. Sci..

[29] Terzi C., Sökmen S., Seçkin S., Albayrak L., Uğurlu M. (2000). Polypoid lesions of the gallbladder: Report of 100 cases with special reference to operative indications. Surgery.

[30] Mellnick V.M., Menias C.O., Sandrasegaran K., Hara A.K., Kielar A.Z., Brunt E.M., Doyle M.B., Dahiya N., Elsayes K.M. (2015). Polypoid lesions of the gallbladder: Disease spectrum with pathologic correlation. Radiographics.

[31] Kimura K., Fujita N., Noda Y., Kobayashi G., Ito K. (2001). Differential diagnosis of large-sized pedunculated polypoid lesions of the gallbladder by endoscopic ultrasonography: A prospective study. J. Gastroenterol..

[32] Cho J.H., Park J.Y., Kim Y.J., Kim H.M., Kim H.J., Hong S.P., Park S.W., Chung J.B., Song S.Y., Bang S. (2009). Hypoechoic foci on EUS are simple and strong predictive factors for neoplastic gallbladder polyps. Gastrointest. Endosc..

[33] Sugiyama M., Atomi Y., Yamato T. (2000). Endoscopic ultrasonography for differential diagnosis of polypoid gall bladder lesions: Analysis in surgical and follow up series. Gut.

[34] Azuma T., Yoshikawa T., Araida T., Takasaki K. (2001). Differential diagnosis of polypoid lesions of the gallbladder by endoscopic ultrasonography. Am. J. Surg..

[35] Cocco G., Basilico R., Delli Pizzi A., Cocco N., Boccatonda A., D’Ardes D., Fabiani S., Anzoletti N., D’Alessandro P., Vallone G. (2021). Gallbladder polyps ultrasound: What the sonographer needs to know. J. Ultrasound.

[36] Lee J.S., Kim J.H., Kim Y.J., Ryu J.K., Kim Y.T., Lee J.Y., Han J.K. (2017). Diagnostic accuracy of transabdominal high-resolution US for staging gallbladder cancer and differential diagnosis of neoplastic polyps compared with EUS. Eur. Radiol..

[37] Jang J.Y., Kim S.W., Lee S.E., Hwang D.W., Kim E.J., Lee J.Y., Kim S.J., Ryu J.K., Kim Y.T. (2009). Differential diagnostic and staging accuracies of high resolution ultrasonography, endoscopic ultrasonography, and multidetector computed tomography for gallbladder polypoid lesions and gallbladder cancer. Ann. Surg..

[38] Kaneko M., Katanuma A., Maguchi H., Takahashi K., Osanai M., Yane K., Hashigo S., Harada R., Kato S., Kato R. (2014). Prospective, randomized, comparative study of delineation capability of radial scanning and curved linear array endoscopic ultrasound for the pancreaticobiliary region. Endosc. Int. Open.

[39] Harvey C.J., Blomley M.J., Eckersley R.J., Cosgrove D.O. (2001). Developments in ultrasound contrast media. Eur. Radiol..

[40] Numata K., Tanaka K., Kiba T., Saito S., Ikeda M., Hara K., Tanaka N., Morimoto M., Iwase S., Sekihara H. (2001). Contrast-enhanced, wide-band harmonic gray scale imaging of hepatocellular carcinoma: Correlation with helical computed tomographic findings. J. Ultrasound Med..

[41] Xu J.M., Guo L.H., Xu H.X., Zheng S.G., Liu L.N., Sun L.P., Lu M.D., Wang W.P., Hu B., Yan K. (2014). Differential diagnosis of gallbladder wall thickening: The usefulness of contrast-enhanced ultrasound. Ultrasound Med. Biol..

[42] Saftoiu A., Napoleon B., Arcidiacono P.G., Braden B., Burmeister S., Carrara S., Cui X.W., Fusaroli P., Gottschalk U., Hocke M. (2020). Do we need contrast agents for EUS?. Endosc. Ultrasound.

[43] Iglesias-Garcia J., Lariño-Noia J., de la Iglesia-García D., Dominguez-Muñoz J.E. (2022). Endoscopic ultrasonography: Enhancing diagnostic accuracy. Best Pract. Res. Clin. Gastroenterol..

[44] Maruyama H., Tobari M., Nagamatsu H., Shiina S., Yamaguchi T. (2022). Contrast-enhanced ultrasonography for the management of portal hypertension in cirrhosis. Front. Med..

[45] Jeong W.K., Kang H.J., Choi S.H., Park M.S., Yu M.H., Kim B., You M.W., Lim S., Cho Y.S., Lee M.W. (2023). Diagnosing Hepatocellular Carcinoma Using Sonazoid Contrast-Enhanced Ultrasonography: 2023 Guidelines From the Korean Society of Radiology and the Korean Society of Abdominal Radiology. Korean J. Radiol..

[46] Xie X.H., Xu H.X., Xie X.Y., Lu M.D., Kuang M., Xu Z.F., Liu G.J., Wang Z., Liang J.Y., Chen L.D. (2010). Differential diagnosis between benign and malignant gallbladder diseases with real-time contrast-enhanced ultrasound. Eur. Radiol..

[47] Zhang H.P., Bai M., Gu J.Y., He Y.Q., Qiao X.H., Du L.F. (2018). Value of contrast-enhanced ultrasound in the differential diagnosis of gallbladder lesion. World J. Gastroenterol..

[48] Negrão de Figueiredo G., Mueller-Peltzer K., Zengel P., Armbruster M., Rübenthaler J., Clevert D.A. (2019). Contrast-enhanced ultrasound (CEUS) and gallbladder diseases—A retrospective mono-center analysis of imaging findings with histopathological correlation. Clin. Hemorheol. Microcirc..

[49] Miwa H., Numata K., Sugimori K., Sanga K., Hirotani A., Tezuka S., Goda Y., Irie K., Ishii T., Kaneko T. (2019). Differential diagnosis of gallbladder polypoid lesions using contrast-enhanced ultrasound. Abdom. Radiol..

[50] Miwa H., Numata K., Sugimori K., Kaneko T., Maeda S. (2021). Vascular evaluation using transabdominal ultrasound for gallbladder polyps. J. Med. Ultrason. 2001.

[51] Zhu L., Han P., Lee R., Jiang B., Jiao Z., Li N., Tang W., Fei X. (2021). Contrast-enhanced ultrasound to assess gallbladder polyps. Clin. Imaging.

[52] Negrão de Figueiredo G., Mueller-Peltzer K., Armbruster M., Rübenthaler J., Clevert D.A. (2019). Contrast-enhanced ultrasound (CEUS) for the evaluation of gallbladder diseases in comparison to cross-sectional imaging modalities and histopathological results. Clin. Hemorheol. Microcirc..

[53] Tamura T., Ashida R., Kitano M. (2022). The usefulness of endoscopic ultrasound in the diagnosis of gallbladder lesions. Front. Med..

[54] Negrão de Figueiredo G., Mueller-Peltzer K., Zengel P., Armbruster M., Rübenthaler J., Clevert D.A. (2018). Diagnostic performance of contrast-enhanced ultrasound (CEUS) for the evaluation of gallbladder diseases1. Clin. Hemorheol. Microcirc..

[55] Negrão de Figueiredo G., Mueller-Peltzer K., Schwarze V., Zhang L., Rübenthaler J., Clevert D.A. (2019). Performance of contrast-enhanced ultrasound (CEUS) compared to MRI in the diagnostic of gallbladder diseases. Clin. Hemorheol. Microcirc..

[56] Franchellucci G., Andreozzi M., Carrara S., De Luca L., Auriemma F., Paduano D., Calabrese F., Facciorusso A., Poletti V., Zerbi A. (2023). Contrast Enhanced EUS for Predicting Solid Pancreatic Neuroendocrine Tumor Grade and Aggressiveness. Diagnostics.

[57] Kitano M., Yoshida T., Itonaga M., Tamura T., Hatamaru K., Yamashita Y. (2019). Impact of endoscopic ultrasonography on diagnosis of pancreatic cancer. J. Gastroenterol..

[58] Yoshida T., Yamashita Y., Kitano M. (2019). Endoscopic Ultrasound for Early Diagnosis of Pancreatic Cancer. Diagnostics.

[59] Ishii T., Katanuma A., Toyonaga H., Chikugo K., Nasuno H., Kin T., Hayashi T., Takahashi K. (2021). Role of Endoscopic Ultrasound in the Diagnosis of Pancreatic Neuroendocrine Neoplasms. Diagnostics.

[60] Hussain A., Weimer D.S., Mani N. (2022). Diagnosing Pancreatic Adenocarcinoma With Contrast-Enhanced Ultrasonography: A Literature Review of Research in Europe and Asia. Cureus.

[61] Ishikawa T., Ohno E., Mizutani Y., Iida T., Koya T., Sasaki Y., Ogawa H., Kinoshita F., Hirooka Y., Kawashima H. (2022). Comparison of contrast-enhanced transabdominal ultrasonography following endoscopic ultrasonography with GD-EOB-DTPA-enhanced MRI for the sequential diagnosis of liver metastasis in patients with pancreatic cancer. J. Hepatobiliary Pancreat. Sci..

[62] Binda C., Coluccio C., Marocchi G., Sbrancia M., Fabbri C. (2021). The Role of Contrast-Enhanced Harmonic Endoscopic Ultrasound in Interventional Endoscopic Ultrasound. Medicina.

[63] Vasilakis T., Ziogas D., Tziatzios G., Gkolfakis P., Koukoulioti E., Kapizioni C., Triantafyllou K., Facciorusso A., Papanikolaou I.S. (2023). EUS-Guided Diagnosis of Gastric Subepithelial Lesions, What Is New?. Diagnostics.

[64] Park C.H., Chung M.J., Oh T.G., Park J.Y., Bang S., Park S.W., Kim H., Hwang H.K., Lee W.J., Song S.Y. (2013). Differential diagnosis between gallbladder adenomas and cholesterol polyps on contrast-enhanced harmonic endoscopic ultrasonography. Surg. Endosc..

[65] Choi J.H., Seo D.W., Choi J.H., Park D.H., Lee S.S., Lee S.K., Kim M.H. (2013). Utility of contrast-enhanced harmonic EUS in the diagnosis of malignant gallbladder polyps (with videos). Gastrointest. Endosc..

[66] Imazu H., Mori N., Kanazawa K., Chiba M., Toyoizumi H., Torisu Y., Koyama S., Hino S., Ang T.L., Tajiri H. (2014). Contrast-enhanced harmonic endoscopic ultrasonography in the differential diagnosis of gallbladder wall thickening. Dig. Dis. Sci..

[67] Sugimoto M., Takagi T., Konno N., Suzuki R., Asama H., Hikichi T., Watanabe K., Waragai Y., Kikuchi H., Takasumi M. (2016). The efficacy of contrast-enhanced harmonic endoscopic ultrasonography in diagnosing gallbladder cancer. Sci. Rep..

[68] Leem G., Chung M.J., Park J.Y., Bang S., Song S.Y., Chung J.B., Park S.W. (2018). Clinical Value of Contrast-Enhanced Harmonic Endoscopic Ultrasonography in the Differential Diagnosis of Pancreatic and Gallbladder Masses. Clin. Endosc..

[69] Numata K. (2015). Advances in ultrasound systems for hepatic lesions in Japan. J. Med. Ultrason. 2001.

[70] Sidhu P.S., Cantisani V., Dietrich C.F., Gilja O.H., Saftoiu A., Bartels E., Bertolotto M., Calliada F., Clevert D.A., Cosgrove D. (2018). The EFSUMB Guidelines and Recommendations for the Clinical Practice of Contrast-Enhanced Ultrasound (CEUS) in Non-Hepatic Applications: Update 2017 (Short Version). Ultraschall Med..

[71] Kawahara S., Tomoda T., Kato H., Ueki T., Akimoto Y., Harada R., Toji T., Okada H. (2022). Accuracy of Endoscopic Transpapillary Gallbladder Drainage with Liquid-Based Cytology for Gallbladder Disease. Digestion.

[72] Inui K., Yoshino J., Miyoshi H. (2011). Diagnosis of gallbladder tumors. Intern. Med..

[73] Matsumori T., Uza N., Okada H., Maruno T., Shiokawa M., Kodama Y., Seno H. (2020). A novel technique for performing gallbladder tumor biopsy using a stent delivery system and biopsy forceps. Endoscopy.

[74] Kobayashi K., Kobara H., Masaki T. (2021). Novel cholangioscopy-guided targeted biopsy for diagnosing gallbladder carcinoma. Dig. Endosc..

[75] Matsumori T., Uza N., Shiokawa M., Maruno T., Nishikawa Y., Morita T., Kuwada T., Marui S., Okada H., Taura K. (2022). Clinical impact of a novel device delivery system in the diagnosis of bile duct lesions: A single-center experience. J. Gastroenterol. Hepatol..

[76] Mandai K., Inoue T., Uno K., Yasuda K. (2022). Transferring a naso-gallbladder drainage tube to the mouth for re-examination of a gallbladder lesion. J. Hepatobiliary Pancreat. Sci..

[77] Yane K., Sumiyoshi T., Kondo H. (2021). Transpapillary gallbladder biopsy using newly designed endoscopic sheath. Dig. Endosc..

[78] Tacelli M., Bina N., Crinò S.F., Facciorusso A., Celsa C., Vanni A.S., Fantin A., Antonini F., Falconi M., Monica F. (2022). Reliability of grading preoperative pancreatic neuroendocrine tumors on EUS specimens: A systematic review with meta-analysis of aggregate and individual data. Gastrointest. Endosc..

[79] Sugimoto M., Irie H., Takagi T., Suzuki R., Konno N., Asama H., Sato Y., Nakamura J., Takasumi M., Hashimoto M. (2020). Efficacy of EUS-guided FNB using a Franseen needle for tissue acquisition and microsatellite instability evaluation in unresectable pancreatic lesions. BMC Cancer.

[80] Levine I., Trindade A.J. (2021). Endoscopic ultrasound fine needle aspiration vs fine needle biopsy for pancreatic masses, subepithelial lesions, and lymph nodes. World J. Gastroenterol..

[81] Rangwani S., Ardeshna D.R., Mumtaz K., Kelly S.G., Han S.Y., Krishna S.G. (2022). Update on endoscopic ultrasound-guided liver biopsy. World J. Gastroenterol..

[82] Varadarajulu S., Eloubeidi M.A. (2005). Endoscopic ultrasound-guided fine-needle aspiration in the evaluation of gallbladder masses. Endoscopy.

[83] Meara R.S., Jhala D., Eloubeidi M.A., Eltoum I., Chhieng D.C., Crowe D.R., Varadarajulu S., Jhala N. (2006). Endoscopic ultrasound-guided FNA biopsy of bile duct and gallbladder: Analysis of 53 cases. Cytopathology.

[84] Goyal S., Prasad G., Chaudhary D., Sakhuja P., Srivastava S., Aggarwal A.K. (2023). Role of Guided FNA in Gallbladder Cancer: A Retrospective 3-Year Study. J. Cytol..

[85] Tong T., Tian L., Deng M.Z., Chen X.J., Fu T., Ma K.J., Xu J.H., Wang X.Y. (2023). The efficacy and safety of endoscopic ultrasound-guided fine-needle biopsy in gallbladder masses. Hepatobiliary Pancreat. Dis. Int..

[86] Singla V., Agarwal R., Anikhindi S.A., Puri P., Kumar M., Ranjan P., Kumar A., Sharma P., Bansal N., Bakshi P. (2019). Role of EUS-FNA for gallbladder mass lesions with biliary obstruction: A large single-center experience. Endosc. Int. Open.

[87] Jacobson B.C., Pitman M.B., Brugge W.R. (2003). EUS-guided FNA for the diagnosis of gallbladder masses. Gastrointest. Endosc..

[88] Kim H.J., Lee S.K., Jang J.W., Kim T.G., Ryu C.H., Park D.H., Lee S.S., Seo D.W., Kim M.H. (2012). Diagnostic role of endoscopic ultrasonography-guided fine needle aspiration of gallbladder lesions. Hepatogastroenterology.

[89] Ogura T., Kurisu Y., Masuda D., Imoto A., Onda S., Kamiyama R., Hayashi M., Mohamed M., Uchiyama K., Higuchi K. (2014). Can endoscopic ultrasound-guided fine needle aspiration offer clinical benefit for thick-walled gallbladders?. Dig. Dis. Sci..

[90] Webber C., Gospodarowicz M., Sobin L.H., Wittekind C., Greene F.L., Mason M.D., Compton C., Brierley J., Groome P.A. (2014). Improving the TNM classification: Findings from a 10-year continuous literature review. Int. J. Cancer.

[91] Seo S., Hatano E., Higashi T., Nakajima A., Nakamoto Y., Tada M., Tamaki N., Iwaisako K., Mori A., Doi R. (2008). Fluorine-18 fluorodeoxyglucose positron emission tomography predicts lymph node metastasis, P-glycoprotein expression, and recurrence after resection in mass-forming intrahepatic cholangiocarcinoma. Surgery.

[92] Kurita A., Kodama Y., Nakamoto Y., Isoda H., Minamiguchi S., Yoshimura K., Kuriyama K., Sawai Y., Uza N., Hatano E. (2016). Impact of EUS-FNA for preoperative para-aortic lymph node staging in patients with pancreatobiliary cancer. Gastrointest. Endosc..

[93] Nagino M., Hirano S., Yoshitomi H., Aoki T., Uesaka K., Unno M., Ebata T., Konishi M., Sano K., Shimada K. (2021). Clinical practice guidelines for the management of biliary tract cancers 2019: The 3rd English edition. J. Hepatobiliary Pancreat. Sci..

[94] Kuraishi Y., Hara K., Haba S., Kuwahara T., Okuno N., Yanaidani T., Ishikawa S., Yasuda T., Yamada M., Fukui T. (2024). Diagnostic performance and safety of endoscopic ultrasound-guided fine-needle aspiration/biopsy for gallbladder lesions. Dig. Endosc..

[95] Facciorusso A., Mohan B.P., Crinò S.F., Ofosu A., Ramai D., Lisotti A., Chandan S., Fusaroli P. (2021). Contrast-enhanced harmonic endoscopic ultrasound-guided fine-needle aspiration versus standard fine-needle aspiration in pancreatic masses: A meta-analysis. Expert. Rev. Gastroenterol. Hepatol..

[96] Tamura T., Yamashita Y., Kawaji Y., Hatamaru K., Itonaga M., Ashida R., Okada K.I., Kawai M., Yamaue H., Kitano M. (2021). Endoscopic ultrasound-guided fine needle aspiration with contrast-enhanced harmonic imaging for diagnosis of gallbladder tumor (with video). J. Hepatobiliary Pancreat. Sci..

[97] Tamura T., Yamashita Y., Itonaga M., Ashida R., Kitano M. (2021). Usefulness of EUS-FNA with contrast-enhanced harmonic imaging for diagnosis of gallbladder tumor. Endosc. Ultrasound.

[98] Rizzato M., Brignola S., Munari G., Gatti M., Dadduzio V., Borga C., Bergamo F., Pellino A., Angerilli V., Mescoli C. (2022). Prognostic impact of FGFR2/3 alterations in patients with biliary tract cancers receiving systemic chemotherapy: The BITCOIN study. Eur. J. Cancer.

[99] Javle M., Borad M.J., Azad N.S., Kurzrock R., Abou-Alfa G.K., George B., Hainsworth J., Meric-Bernstam F., Swanton C., Sweeney C.J. (2021). Pertuzumab and trastuzumab for HER2-positive, metastatic biliary tract cancer (MyPathway): A multicentre, open-label, phase 2a, multiple basket study. Lancet Oncol..

[100] Hirata K., Kuwatani M., Suda G., Ishikawa M., Sugiura R., Kato S., Kawakubo K., Sakamoto N. (2019). A Novel Approach for the Genetic Analysis of Biliary Tract Cancer Specimens Obtained Through Endoscopic Ultrasound-Guided Fine Needle Aspiration Using Targeted Amplicon Sequencing. Clin. Transl. Gastroenterol..

[101] Chan H.P., Samala R.K., Hadjiiski L.M., Zhou C. (2020). Deep Learning in Medical Image Analysis. Adv. Exp. Med. Biol..

[102] Chen X., Wang X., Zhang K., Fung K.M., Thai T.C., Moore K., Mannel R.S., Liu H., Zheng B., Qiu Y. (2022). Recent advances and clinical applications of deep learning in medical image analysis. Med. Image Anal..

[103] Zou J., Gao B., Song Y., Qin J. (2022). A review of deep learning-based deformable medical image registration. Front. Oncol..

[104] Hermsen M., de Bel T., den Boer M., Steenbergen E.J., Kers J., Florquin S., Roelofs J., Stegall M.D., Alexander M.P., Smith B.H. (2019). Deep Learning-Based Histopathologic Assessment of Kidney Tissue. J. Am. Soc. Nephrol..

[105] Jungo A., Scheidegger O., Reyes M., Balsiger F. (2021). pymia: A Python package for data handling and evaluation in deep learning-based medical image analysis. Comput. Methods Programs Biomed..

[106] Jiang H., Diao Z., Shi T., Zhou Y., Wang F., Hu W., Zhu X., Luo S., Tong G., Yao Y.D. (2023). A review of deep learning-based multiple-lesion recognition from medical images: Classification, detection and segmentation. Comput. Biol. Med..

[107] Jiang W., Zeng G., Wang S., Wu X., Xu C. (2022). Application of Deep Learning in Lung Cancer Imaging Diagnosis. J. Healthc. Eng..

[108] Akkus Z., Cai J., Boonrod A., Zeinoddini A., Weston A.D., Philbrick K.A., Erickson B.J. (2019). A Survey of Deep-Learning Applications in Ultrasound: Artificial Intelligence-Powered Ultrasound for Improving Clinical Workflow. J. Am. Coll. Radiol..

[109] Le E.P.V., Wang Y., Huang Y., Hickman S., Gilbert F.J. (2019). Artificial intelligence in breast imaging. Clin. Radiol..

[110] Gupta R., Srivastava D., Sahu M., Tiwari S., Ambasta R.K., Kumar P. (2021). Artificial intelligence to deep learning: Machine intelligence approach for drug discovery. Mol. Divers..

[111] Anwar S.M., Majid M., Qayyum A., Awais M., Alnowami M., Khan M.K. (2018). Medical Image Analysis using Convolutional Neural Networks: A Review. J. Med. Syst..

[112] Zhou S.K., Greenspan H., Davatzikos C., Duncan J.S., van Ginneken B., Madabhushi A., Prince J.L., Rueckert D., Summers R.M. (2021). A review of deep learning in medical imaging: Imaging traits, technology trends, case studies with progress highlights, and future promises. Proc. IEEE Inst. Electr. Electron. Eng..

[113] Obaid A.M., Turki A., Bellaaj H., Ksantini M., AlTaee A., Alaerjan A. (2023). Detection of Gallbladder Disease Types Using Deep Learning: An Informative Medical Method. Diagnostics.

[114] Jang S.I., Kim Y.J., Kim E.J., Kang H., Shon S.J., Seol Y.J., Lee D.K., Kim K.G., Cho J.H. (2021). Diagnostic performance of endoscopic ultrasound-artificial intelligence using deep learning analysis of gallbladder polypoid lesions. J. Gastroenterol. Hepatol..

[115] Jeong Y., Kim J.H., Chae H.D., Park S.J., Bae J.S., Joo I., Han J.K. (2020). Deep learning-based decision support system for the diagnosis of neoplastic gallbladder polyps on ultrasonography: Preliminary results. Sci. Rep..

